# An analytical characterization study on biofuel obtained from pyrolysis of *Madhuca longifolia* residues

**DOI:** 10.1038/s41598-024-65393-7

**Published:** 2024-06-26

**Authors:** S. Thiru, Ramesh Kola, Manish Kumar Thimmaraju, C. Sowmya Dhanalakshmi, Vipin Sharma, P. Sakthi, Lakshmana Phaneendra Maguluri, L. Ranganathan, J. Isaac JoshuaRamesh Lalvani

**Affiliations:** 1 Department of Mechanical and Materials Engineering, University of Jeddah, 23218 Jeddah, Kingdom of Saudi Arabia; 2 Department of Chemistry, Chaitanya Bharathi Institute of Technology (A), Gandipet, Hyderabad, Telangana 500075 India; 3 Department of Pharmaceutical Analysis, Balaji Institute of Pharmaceutical Sciences, Narsampet, Warangal, Telangana 506331 India; 4 Department of Mechanical Engineering, SNS College of Technology, Coimbatore, Tamil Nadu 641035 India; 5 Department of Mechanical Engineering, Medi-Caps University, Indore, Madhya Pradesh 453331 India; 6 Department of Electronics and Communication Engineering, M.Kumarasamy College of Engineering, Karur, Tamil Nadu 639113 India; 7 Department of Computer Science and Engineering, Koneru Lakshmaiah Education Foundation, Vijayawada, Andhra Pradesh 522302 India; 8 Department of Mechanical Engineering, Cambridge Institute of Technology, Tatisilwai, Ranchi, Jharkhand 835103 India; 9 Faculty of Mechanical Engineering, Arba Minch Institute of Technology, Arba Minch University, PO Box 21, Arba Minch, Ethiopia

**Keywords:** Forest residues, Pyrolysis, Fixed bed, FT-IR, GC–MS, Environmental impact, Crop waste

## Abstract

The current study focuses on examining the characteristics of biofuel obtained from the pyrolysis of *Madhuca longifolia* residues, since the selected forest residue was primarily motivated by its greater volatile matter content. The study used several analytical techniques to describe pyrolysis oil, char, and gas obtained from slow pyrolysis process conducted between 350 and 600 °C in a fixed-bed reactor. Initially, the effect of process temperature on product distribution was assessed to motivate maximum pyrolysis oil yield and found to be 44.2 wt% at pyrolysis temperature of 475 °C, while the yields of char and gas were 22.1 wt% and 33.7 wt%, respectively. In order to determine the suitability of the feedstock, the *Madhuca longifolia* residues were analyzed by TGA and FT-IR, which revealed that the feedstock could be a feasible option as an energy source. The characterization of pyrolysis oil, char, and gas has been done through various analytical methods like FT-IR, GC-MS, and gas chromatography. The physicochemical characteristics of the pyrolysis oil sample were examined, and the results showed that the oil is a viscous liquid with a lower heating value than conventional diesel. The FT-IR and GC-MS analysis of pyrolysis oil revealed the presence of increased levels of oxygenated chemicals, acids, and phenol derivatives. The findings of the FT-IR analysis of char indicated the existence of aromatic and aliphatic hydrocarbons. The increased carbon content in the char indicated the possibility of using solid fuel. Gas chromatography was used to examine the chemical structure of the pyrolysis gas, and the results showed the existence of combustible elements.

## Introduction

The increased residents and transportation in the world is experiencing a need for huge amounts of energy and the repercussions of global warming. Meanwhile, the use of petroleum-based fuels is also increasing on a daily basis. Globally, energy output has significantly increased recently in order to equate the demand for conventional fuels^1^. The carbon neutrality of biofuel possesses huge potential for reducing carbon emissions and existing fossil fuel dependence. Emissions of carbon can be effectively decreased by using alternative fuels. Burning fossil fuels is closely linked to a number of serious health issues. Burning fossil fuels for energy causes pollution in the air and water, which has an adverse effect on the surroundings. Sustainable energy is energy that can satisfy current energy demands without endangering future energy supplies and resources^2^. Wind, solar, bioenergy, and hydroelectric are the four most important sustainable energy sources. Renewable organic material derived from plants and animals is called biomass. Biomass is an important fuel for cooking and heating. It can be burned directly to provide heat or converted to liquid and gaseous fuels via various bio- and thermochemical conversion processes^3^. Various low- and medium-cost agricultural and biological wastes can be transformed into biofuels. In comparison to biological techniques, thermochemical technologies offer much faster reaction times and greater feedstock flexibility. The three primary process techniques that can be employed to transform biomass into biofuel are physiochemical, thermochemical, and biochemical. Fermentation and anaerobic digestion are considered biochemical conversion processes. Pyrolysis, gasification, and combustion are considered thermochemical conversion processes. Compared to biochemical conversion processes, thermochemical conversion of biomass into biofuel is the most favorable method to provide energy for the future^4^. In biomass thermal conversion processes, biomass is broken down by applying heat, usually above 300 °C, in order to convert it into different types of energy, such as power, heat, or biofuels. The physical characteristics of the resulting biofuel depend on the presence of numerous components. A recent study represented that, out of the 181.5 billion tonnes of lignocellulosic wastes, only 4.5% is utilized each year^5^. Biofuel has primarily been produced using non-edible, oil-rich seeds and agro-forestry biomass wastes. However, any kind of biomass can be turned into biofuel through thermal cracking or pyrolysis.

In India, almost 22% of the land is covered with natural forest. *Madhuca longifolia*, a tree native to India, has remarkable potential. The tree is primarily cultivated on the Indo-Pakistan subcontinent. It belongs to the Sapotaceae family and is considered an Indian forest plant. The tree is abundant in Indian forests. Since ancient times, tribal peoples in India have cultivated this tree for various applications, including food, fuel, and fertilizer. The various parts and their numerous applications of the tree are also summarized in the literature^6^. Previously, many parts of the *Madhuca longifolia* tree have been utilized for producing biofuel. Mishra and Mohanty^7^ concentrated on the catalytic pyrolysis of seeds obtained from *Madhuca longifolia*. The authors conducted pyrolysis experiments with and without catalysts in a batch-tyre reactor and produced 51.2 wt% of pyrolysis oil. In this study, the utilization of catalysts reduced the yield of oil. Raj et al.^8^ used *Madhuca longifolia* wood and low-grade coal to produce producer gas using the co-gasification process and utilized it as a fuel for IC engines. The study found that the co-gasification system provides a viable solution for alternate power generation and may prove beneficial for small-scale enterprises. Shanmuga Priya and Rajalakshmi^9^ used pyrolysis and hydrothermal methods to produce functional carbon material from *Madhuca longifolia* leaves. In order to improve their internal properties, the produced carbon materials were further activated. Several analytical methods, including Fourier transform infrared spectroscopy (FT-IR) and scanning electron microscopy, were used to analyze the produced carbon materials and found that they can be used as environmentally friendly, low-cost electrode materials for supercapacitors.

Pyrolysis is a useful method for turning low-value biomass and biobased wastes into biofuel. It is a highly effective technique for turning biomass into a liquid intermediary that can be further processed into hydrocarbon biofuels. Pyrolysis is the key technology of biobased power generation in which solid biomass is converted into liquid, solid, and gaseous compounds. Pyrolysis oil is the combination of water and different organic molecules^10^. The pyrolyzed oils are used in multiple ways as low-grade fuels for furnaces and have a lot of possible ways to separate chemicals via cracking, hydrogenation and aqueous phase processing. It is also more important to select a proper biomass feedstock that will enable the requisite heat transfer rates to produce more pyrolysis oil yields. The selection of feedstock plays a significant role in achieving a better yield. Typically, land biomass wastes such as wood, wood bark, plant residue and shells are commonly used feedstocks. In some cases, microalgae also served as a beneficial feedstock. Other than the feedstocks characteristics, numerous factors, including reactor type, feedstock size and process time of the feedstock and volatile matters, also impact the yield of the biofuel^11^. The most popular reactors for slow pyrolysis are drums, rotatory kilns and screw types. On the other side, fluidized beds, ablative reactor, vortex reactor and rotating disk reactors are suitable for fast pyrolysis. Biomass is a sustainable alternative source of renewable energy. Cellulose, hemicellulose and lignin are the basic natural polymeric materials present in biomass and extractives and minerals are the other two components present within the lignocellulosic material which distributes in different ratios^12^. Previously, many authors have conducted pyrolysis reactions using different biomass residues in different reactors and their product yield under optimum conditions are displayed in Table 1.

**Table 1:** Pyrolysis of different biomass residues and product yields.

Biomass type	Reactor type	Temperature in °C	Particle size in mm	Heating rate in °C/min	Pyrolysis product yield in wt%			Reference
					Pyrolysis oil	Char	Gas	
Rice husk	Fixed bed	450	0.5–2.0	20	38.1	35.0	26.9	^13^
Wheat straw	Fixed bed	400	0.5–2.0	20	36.7	34.4	28.9	^13^
Cashew nut shell	Batch	400	0.25	22.5	40.0	30.0	30.0	^14^
*Xanthium strumarium*	Fixed bed	450	0.15–0.22	50	22.7	32.2	45.0	^15^
*Calophyllum inophyllum* shell	Fixed bed	425	425	40	41.0	#	#	^16^
Eremurus spectabilis	Fixed bed	500	0.22–0.85	50	34.6	#	#	^17^
Cotton stalk	Fixed bed	600	#	20	17.1	38.0	44.8	^18^
Napier grass stem	Fixed bed	500	0.2–2.0	30	32.2	#	#	^19^
*Anchusa azurea*	Fixed bed	450	0.6	100	31.3	37.6	31.2	^20^
Eastern giant fennel stalks	Fixed bed	500	0.15–0.85	50	45.2	24.3	30.4	^21^
Rice straw	Fixed bed	500	#	10	43.3	28.0	–	^22^
*Giant miscanthus*	Fixed bed	550	#	10	50.7	26.2	23.2	^23^
Babool seeds	Fixed bed	450	0.4–1.0	25	38.3	#	#	^24^
Switchgrass	Fixed bed	600	2.0	#	37.0	26.0	35.0	^25^
Mustard de-oiled cake	Fixed bed	550	#	25	53.2	29.9	16.7	^26^
Napier grass	Fixed bed	500	1.0–2.0	150	36.0	30.0	#	^27^
Olive residue	Fixed bed	500	1.29	7	39.0	#	#	^28^
Pine forest residues	Auger reactor	500	20.0	#	59.0	26.0	#	^29^

^#^Not reported.

The 3Rs—reduce, reuse, and recycle—contribute to long-term environmental development. Because forestry wastes are abundant in biomaterials, if employed, they might become the backbone of sustainable development, producing a variety of opportunities for future generations^30^. Carrasco et al.^31^ carried out a pyrolysis test using Maine forest residues. The authors produced 61, 24 and 15 wt% of oil, char and gas. The total yield was predicted roughly up to 16% by mass and 40% by energy. Amutio et al.^32^ studied the feasibility of valorization of different forest residues, specifically bushes in a conical reactor. The reactor was set to 500 °C and the material utilized in this research yielded 80 wt% of oil with a maximum of 23 wt% char and 5 wt% gas fractions. The characterization study of the pyrolysis oil showed 35 wt% of water molecules in the oil, with a majority of phenols, ketones, acids, and furans. Several precise characterization approaches were used to find the information about biofuels. For characterization, the majority of previous pyrolysis studies dealt with FT-IR, Gas chromatography Mass Spectroscopy (GC–MS) or a combination of these. The spectroscopic techniques provide extremely significant details about its functional groups and can aid in the documentation of yields. Identifying the physiochemical nature of the pyrolysis biofuel is also important to understand the industrial applications of the products. Charon et al.^33^ developed various analytical techniques to analyze six bio-oils obtained from hardwood, softwood and wheat grass. The result showed that the wood based pyrolysis oils were single-phase liquids, whereas the wheat grass pyrolysis oil was heterogeneous. The chromatography analysis of pyrolysis oil showed more than 100 elements. Schellekens et al.^34^ utilized GC/MS to explore molecular characteristics of biofuels obtained from different agricultural residues. They discovered that, regardless of pyrolysis feedstocks, the process temperature is an important factor that determines yield compositions. They also showed that the higher molecular weight products of all chemicals in char decreased as the reaction temperature increased.

The current study provides light on the characterization of the biofuels derived from *Madhuca longifolia* residues collected from mature trees. Pyrolysis of the collected residues was executed in a fixed bed reactor by changing the temperature from 350 to 600 °C, since this operating temperature yielded the maximum pyrolysis oil. The pyrolysis oil obtained under the maximum yield conditions was evaluated using various analytical methods and its physicochemical parameters were also assessed based on ASTM standards. Apart from pyrolysis oil, the char and pyrolysis gas were also examined to find their components. The key aim of the study was to define the subfractions acquired from the pyrolysis of *Madhuca longifolia* residues analytically and to determine their potential for commercial applications, since most of the previous studies concentrated on the utilization of wood, bark, seeds, seed cake, and leaves of *Madhuca longifolia*, no studies were identified on the utilization of residues for the production of biofuel via pyrolysis process. This study utilized various analytical techniques, including FT-IR, GC–MS, and gas chromatography, to characterize the produced biofuels. Furthermore, the pyrolysis oil was examined in its natural form to identify its basic components.

## Materials and methods

### *Madhuca longifolia* residues

*Madhuca longifolia* residues were collected from the west part of Tamil Nadu, India. The residues are a mixture of wood, wood bark, leaves and roots obtained from a single mature tree. The feedstocks were separated manually from other types of wood materials and washed with fresh water. Before starting the pyrolysis experiment, the moisture present in the samples was reduced by natural and vacuum drying. The collected residues were first ground in a ball mill (Laxmi Engineers, Rajasthan, India), and then sieved using a sieve shaker (Jayant Test Sieves, Sunshine Instruments, Coimbatore, India) to have an average diameter of less than 1.0 mm. The screened particles were dried naturally in an open sun for 2 weeks. Again, the naturally dried particles were kept in a furnace for 24 h, maintained at a temperature of 100 °C. For experimentation, the dried samples were stored in a tight container.

### Pyrolysis reactor and procedure

**Figure 1:**
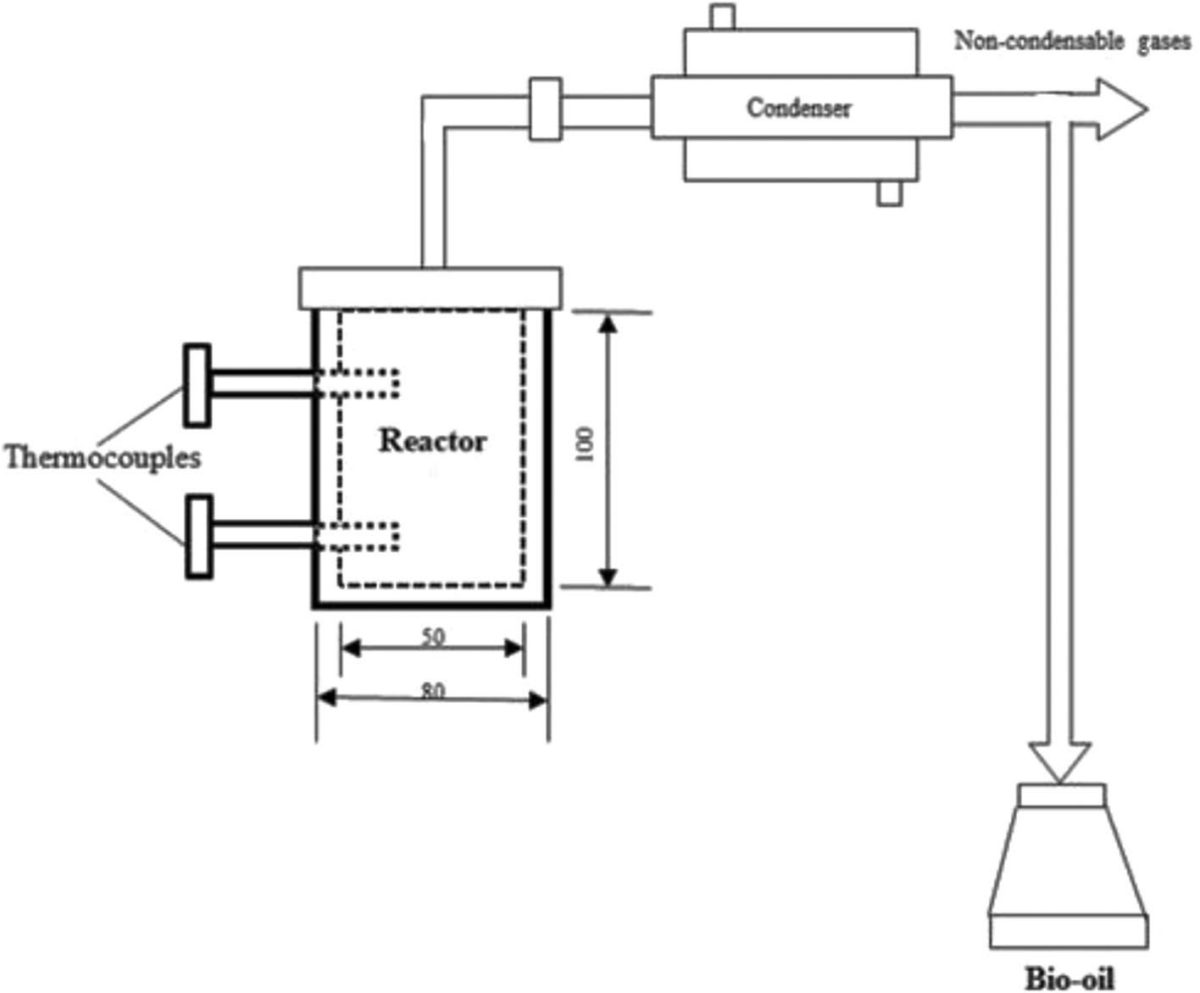
Experimental set up.

The pyrolysis tests on *Madhuca longifolia* residues were conducted in a fixed-bed reactor. The reactor is made up of a cylindrical heater. It has 50 mm internal diameter and 100 mm height. For each experiment, 60 g of feedstock are kept inside the reactor. The reactor was heated by an external 2 kW cylindrical heater equipped with an ammeter and voltmeter setup. Pyrolysis experiments were conducted at temperatures ranging from 350 to 600 °C. For this, the heating rate was set to 20 °C/min. In order to measure the gas phase temperature of the reactor, two K-type thermocouples were provided inside the reactor at two different points. The desired temperature was attained using a PID controller coupled to a furnace via an autotransformer. The setup had a top-opening system to release condensable volatiles. The volatile compounds released from the reactor are transferred into the volatile recovery unit, or condensing unit, where the condensable vapours are separated from the non-condensable gases. Volatile vapours are rapidly quenched inside the condenser, which prevents the subsequent reactions that turn condensable volatiles into permanent or non-condensable gases. In order to get maximum pyrolysis oil conversion, till the reactor reaches the atmospheric temperature, the condenser is supplied with an adequate quantity of ice water maintained at 5 °C. Inside the condenser, when the pyrolysis gas reaches 50 °C, the condensable pyrolysis oil and water usually begin to condense, whereas the phenolic compounds will begin to condense at 80 °C. It should be noted that the partial pressure of vapour compounds is also a function of composition, which is influenced by the type of feedstock and reactor operating conditions^35^. The condensed liquid, called pyrolysis oil, can be collected in the jar kept at the bottom of the condenser. Figure 1 illustrates the reactor system. The current experimental setup that could have an impact on the pyrolysis yields is displayed in Table 2. In order to find the repeatability, each run was conducted three times, and the average was taken for the analysis.

**Table 2:** Experimental condition.

Case	Reactor temperature in °C	Particle size in mm	Feeding rate in grams
Run 1	350	1.0	60
Run 2	375	1.0	60
Run 3	400	1.0	60
Run 4	425	1.0	60
Run 5	450	1.0	60
Run 6	475	1.0	60
Run 7	500	1.0	60
Run 8	525	1.0	60
Run 9	550	1.0	60
Run 10	575	1.0	60
Run 11	600	1.0	60

### Characterization of feedstock and pyrolysis products

#### Proximate analysis

The proximate analysis of any biofuel or feedstock describes the volatile matter content (ASTM D3175), amount of moisture (ASTM D3173), ash (ASTM D3174) and fixed carbon. The analysis in this study on biomass samples was conducted in a muffle furnace according to the above mentioned ASTM standards with good temperature control and a good weight balancing machine (± 0.1 mg sensitivity). The formula used for the proximate analysis is given below.

1 $$\%M= \frac{weight\,of\,sample\,before\,drying-weight\,of\,sample\,after\,drying}{weight\,of\,sample\,before\,drying} \times 100$$

2 $$\%VM= \frac{oven-dried\,sample\,weight-weight\,of\,sample\,remaining\,after\,burning}{oven-dried\,sample\,weight} \times 100$$

3 $$\%A \left(air-dried\,sample\right)=\left(\frac{{W}_{1}}{{W}_{2}\times \frac{T}{100}}\right)\times 100$$

where $${W}_{1}=weight\,of\,ash$$, $${W}_{2}=initial\,weight\,of\,sample$$, $$T=percent\,of\,total\,solid$$.

4 $$\%FC=100-(\%M+\%VM+\%A)$$

#### Ultimate and lignocellulosic analysis

The ultimate analysis identifies the percent of carbon (C), hydrogen (H), nitrogen (N), sulfur (S), and oxygen (O) in the biomass sample and produces char according to the prescribed ASTM standards. This analysis yields the composition on ash-free basis. The analysis was carried out by using an elemental analyzer (EA 2400 Series II). The CHNS mode is based on the conventional PreglDumas technique, which involves burning samples in an atmosphere with just oxygen and collecting the combustion gases as a byproduct^36^. For the analysis, a small amount of samples were fully burned and reduced to the constituent gases CO_2_, H_2_O, N_2_, and SO_2_ in the presence of oxygen and combustion reagents. The presence of carbon in the sample turns CO_2_, while H_2_ to H_2_O, N_2_ to NOx, and S to SO_2_. After being passed over a heated, high-purity copper surface, the gases exiting the combustion chamber lose all oxygen and turn any NOx into N_2_. The identification of the production of CO_2_, H_2_O, N_2_, and SO_2_ gases yields the values of C, H, N, and S. The combusted gases are passed through a high-quality copper surface, which eliminates all oxygen and turns any NOx into N_2_. The identification of the production of CO_2_, H_2_O, N_2_, and SO_2_ gases yields the values of C, H, N, and S.

The lignocellulosic content of the biomass samples is measured using the conventional wet chemistry method. This method is considered an effective one for the determination of the lignocellulosic content of any biomass material. The analysis was performed by consuming 0.5 g of biomass. The analysis was initiated by acid chlorination treatment using NaClO_2_ and CH_3_COOH combinations. The treatment was performed at a temperature of 75 °C. After that, more NaClO_2_ and CH_3_COOH are continuously added for successive cycles of chlorination. After filtration, the resulting solution is then cleaned with acetone and normal water. Cellulose and hemicellulose were found at the end of the filtration process, and the amount of lignin in the sample was determined by a two-step sulfuric acid hydrolysis process.

#### Thermogravimetric analysis

Thermogravimetric analysis (TGA) and derivative thermogravimetry (DTG) has been extensively utilized to explore thermal processes and kinetics of any organic material. It measures the mass loss of the material regarding temperature and time. Analysis of TGA helps in the preparation, design, and process of the industrial pyrolysis system^37^. The TGA study provides extensive experimental data regarding the pyrolysis performance of biomass. The analysis was carried out using a TGA analyzer (TGA701) by heating the sample to 600 °C at a heating rate of 20 °C/min.

#### FT-IR analysis

FT-IR quantification techniques for volatile compounds relevant to pyrolysis have been established by many literature in recent years. FT-IR spectroscopy was used to analyze the microstructures of feedstock, pyrolysis oil and char products. For the analysis, the potassium bromide (KBr) disk approach was used to prepare the samples. The compressed alkali metal halide pellet method, also known as the KBr pellet or disk method, is a widely used technique for handling solid samples^38^. The KBr disk approach is a valuable method in IR spectrometry. In this method, the samples are converted into powdered form and combined with an IR transparent salt, like KBr, to lower the sample's concentration and improve the spectrum. To eliminate water molecules from the KBr, it is pulverized into 200 mesh sizes and dried at 110 °C. For the preparation of the pellet, 200 mg of powdered KBr is blended with a 1% sample, and the combined sample is then pressed into the disk. The spectra were acquired using a Bruker Tensor 27 spectrometer (Bruker, Germany) with 4 cm^−1^ resolution between 4000 and 400 cm^−1^.

#### GC–MS analysis

Thermo GC—trace ultra-version: 5.0, Thermo MS DSQ II supplied by Thermo Scientific Corporation was used to conduct GC–MS analysis of the oil. Quartz wool was placed at one end of a pre-weighed quartz tube, which had a 25 mm length and 0.5 mm ID and samples weighing 0.5 mg were placed for the analysis. The split injector of the GC inlet port was set to 280 °C and a split ratio of 20:1 was selected for analysis. A DB 35-MS capillary standard non-polar column of length 30 m, diameter 0.25 mm and 0.25 μm film thickness was used for the separation of pyrolysis yields. The temperature program was set to 40 °C, held for 2 min and then ramped to 280 °C at a rate of 6 °C/min. The interface temperature was set to 280 °C and the mass spectrometer's ion source to 230 °C, and the scanning was performed in the range of 50–550 *m/z*.

#### Physical characterization of pyrolysis oil

The various physical characteristics of the pyrolysis oil were found by following different ASTM protocols. To find viscosity, a Redwood viscometer (Model: SICBRV-01, Shambahavi Imp., Mumbai) was used. The density was found by weighing known volume. The Scientech supplied Cleave Land Flash Point Kit (Model SE-224) was utilized to measure the flash point..

#### Determination of heating value

The higher heating value of the pyrolysis products was measured on a Parr-6772 (Parr Instrument Company, Illinois, USA) apparatus according to ASTM D240 protocol. The values displayed are calculated with 0.6% accuracy and are based on the average of several experiments.

#### Gas chromatography

With the use of a Shimadzu gas analyzer (Model: GC-2014, Shimadzu Corporation), the produced gas obtained at 475 °C was examined to find its gas sub-fractions. The analysis was done using a splitless injection unit armed with a micropack with a carbon column of 1 mm diameter. The accuracy of the chromatogram was ± 1% (0.01 °C) the range of the thermal conductivity detector was 400 °C and the linear heating range was set to 20 °C/min up to 250 °C.

### Ethics statement

All experimental and laboratory tests were performed in accordance with the relevant guidelines and regulations.

### Permission to collect biomass residue

The residues used for this study were collected from plants on private lands and we obtained permission from the landowner to access the areas and collect the residues.

## Results and discussion

### Characterization of feedstock

#### Proximate and ultimate analysis

The elemental analysis of the feedstock is found as follows: C = 47.2%, H = 5.9%, N = 3.6%, S = 1.2% and O = 42.1%. C and O are available more than H and N. When biomass is pyrolyzed, the higher concentration of O content results in oxygenated products. Lower levels of S and N suggest that during pyrolysis, it releases fewer SOx and NOx. The empirical formula derived from elemental analysis is CH_1.489_N_0.065_O_0.669_. It is well known that a material having lower H/C and O/C ratio have higher energy content^39^. The proximate analysis shows lower ash and moisture levels. Initially, the moisture level was more, but it was reduced by the continuous drying process. For pyrolysis, the biomass must have a moisture content of less than 10%^40^. The selection of appropriate biomass material for pyrolysis is the key process. The physicochemical analysis of the present biomass reported in Table 3 shows its suitability. The selected residue has a volatile matter content of 71.26%. Typically, biomass with a higher volatile matter content yields large amounts of biogas and oil^41^. The yield of pyrolysis products increases directly proportional to the amount of volatile materials extracted. Almost 90% of the volatile content in the feedstock is lost during pyrolysis to produce biofuels. Biomass with a high level of volatile content is generally chosen for pyrolysis since it is more reactive and readily devolatilized. For the pyrolysis process, the amount of ash in biomass should be minimal^42^. The ash content of the residue is 4.50%, which is lower than coal, switchgrass, barley straw and wheat straw mentioned in the literature^39^. The yield of the pyrolysis products and process efficiency are directly impacted by the ash present in the feedstock. So the ash content in the feedstock is an important parameter for the pyrolysis process. Yildiz et al.^43^ reported that adding ash to the pyrolysis process resulted in higher amounts of non-condensable gases and water while decreasing the yields of pyrolysis oil. The cellulose, hemicellulose and lignin contents are 18.04, 45.18 and 32.78% respectively. From proximate and ultimate analysis, it can be understood that the selected feedstock is suitable for the production of biofuel. Table 3 indicates the result of proximate, ultimate analysis of *Madhuca longifolia* residues.

**Table 3:** Proximate and ultimate analysis of *Madhuca longifolia* residues.

Parameter	Value in wt%
Proximate analysis	
Moisture	5.98
Ash	4.50
Volatile matter	71.26
Fixed carbon^a^	18.24
Ultimate analysis (dry ash basis)	
C	47.2
H	5.9
N	3.6
S	1.2
O^a^	42.1
H/C molar ratio	1.489
O/C molar ratio	0.669
Empirical formula	CH_1.489_N_0.065_O_0.669_
HHV (MJ/kg)	17.54
Component analysis	
α-Cellulose	18.04
Hemicellulose	45.18
Lignin	32.78

^a^By difference.

#### TGA and DTG analysis

**Figure 2:**
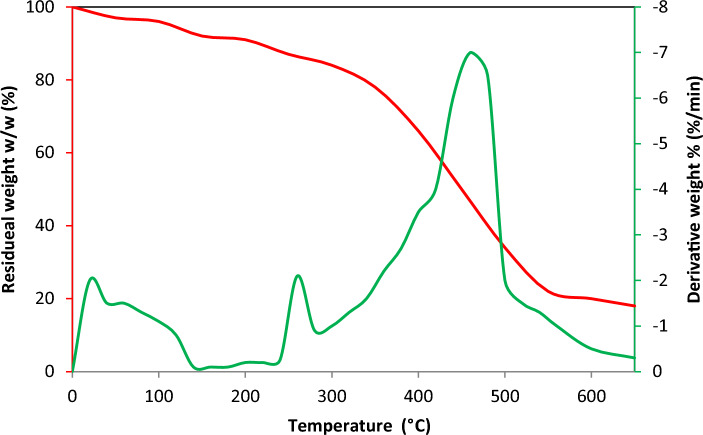
TGA and DTG analysis of *Madhuca longifolia *residue.

For the pyrolysis process, finding thermal behavior of the feedstock is important before conducting the experimental process. The TGA and DTG analysis of the *Madhuca longifolia* residues are revealed in Fig. 2. The figure indicates the results of the DTG curves at a 20 °C/min heating rate. The decomposition of the sample may be affected by differences in heat transfer and kinetic rates. It can be found that heating rates primarily influence the stages of pyrolysis. Increasing heating rates alter the maximum weight loss point at different temperature ranges^44^. The peaks observed in DTG represent the point or temperature at which the rate of weight loss reaches its maximum. In an inert atmosphere, the solid heats up in three different regimes. In the first regime, only heating takes place, followed by pyrolysis in the second regime, and further heating in the third regime if there is char remaining. The heat applied to the biomass breaks down its constituents. It is the process of decomposing a chemical compound into smaller components through various chemical reactions. Here, the mass loss function of residues is detected in three phases. The first phase represents the evaporation of moisture, in which ⁓ 12% of mass loss occurs due to the removal of moisture at < 140 °C. Furthermore, two exothermic peaks can be seen in the analysis at 245 °C and 457 °C. Between 345 °C and 520 °C a maximum mass loss of ⁓ 75% was observed. Additionally, the DTG curve for this stage reveals a peak at 457 °C with a maximum mass loss rate of 1.22 mg/min. Mass loss during this stage are linked to the disintegration of hemicellulose, which represents active pyrolysis, and the disintegration of cellulose and lignin, which represents active and passive pyrolysis^45^. Notably, the sample displayed its largest weight loss at its peak temperature, indicating a high degree of thermal degradation reactivity. The results of this study are in line with those of Dwivedi et al.^44^. Around 450 °C, all parts of the biomass material break down extremely and lose their maximum weight. The weight loss differential decreases after 460 °C and reaches zero after 600 °C. For last stage above 600 °C, the degradation of biomass decreases and almost same after that. The residues that remained at the end of the TGA analysis represent the presence of ash. The percentage of ash, which may be computed at around 5% by weight, is the quantity of material that remains after reaching 600 °C.

#### FT-IR analysis

**Figure 3:**
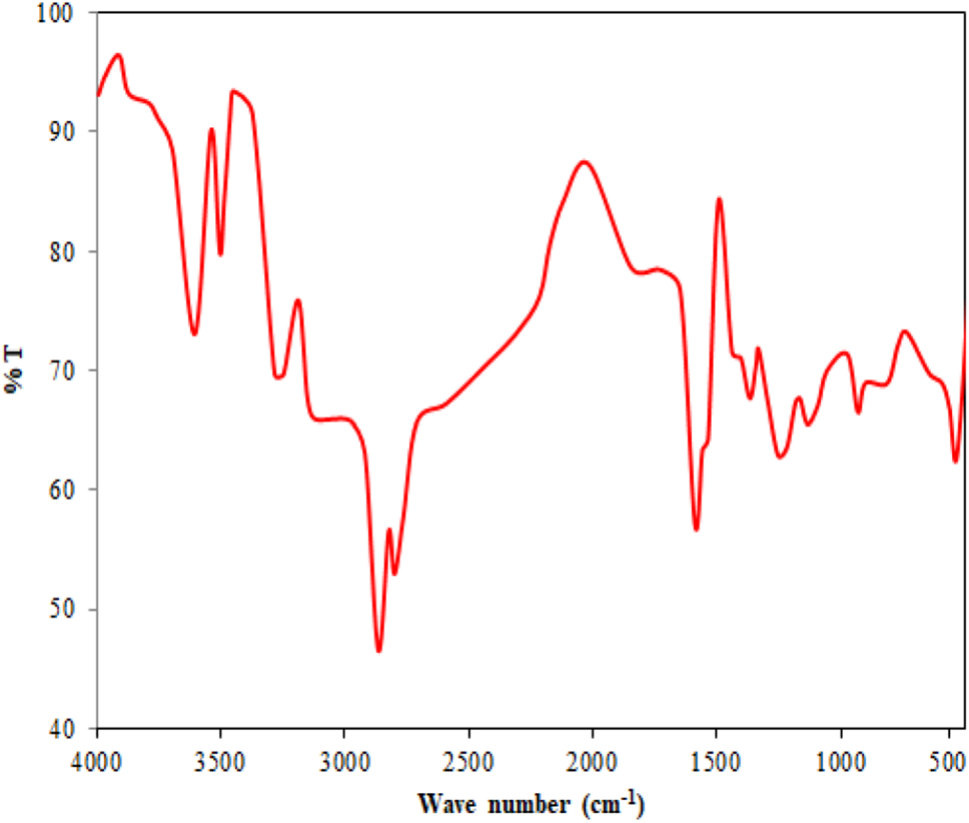
FT-IR analysis of *Madhuca longifolia *residue.

The peaks, which are also called absorbance bands, match with the various vibrations of the sample’s atoms when it is contacted in the infrared region of the electromagnetic spectrum. The x-axis in the spectra denotes the infrared spectrum, and the y-axis denotes the quantity of infrared light transmitted or absorbed by the sample. In the spectra, the peaks are attributable to the specific functional group. A greater concentration of the appropriate functional group or bond is indicated by a stronger absorption peak. Figure 3 illustrates FT-IR analysis of *Madhuca longifolia* residue. The figure shows plots between wavenumber and transmittance spectra. The existence of water, phenolic compounds, aromatic and other impurities in the feedstock was confirmed by the peak at 3605.5 cm^−1^ which was linked to the O–H group^46^. The possible existence of alkanes was suggested by the adsorption bond 2865.6 cm^−1^, which was linked to the C–H stretching vibration^47^. A C≡C deformation-attributed adsorption bond at 1402.9 cm^−1^ revealed the existence of alkynes^48^, whereas an adsorption bond at 1610.3 cm^−1^ associated with C=O stretching vibration indicated the existence of aldehyde, ketones, or carboxylic acids^7^. Alkanes were identified by the peak 1368.1 cm^−1^ ascribed to C–H bending and the existence of ethers, alcohols, and carboxylic acid was indicated by the adsorption band 1217.9 cm^−1^ attributable to C–O bending. The identification of O–H bending stretching is responsible for the existence of mono- and polycyclic aromatic compounds between 931.4 cm^−1^.

### Pyrolysis product yields

**Figure 4:**
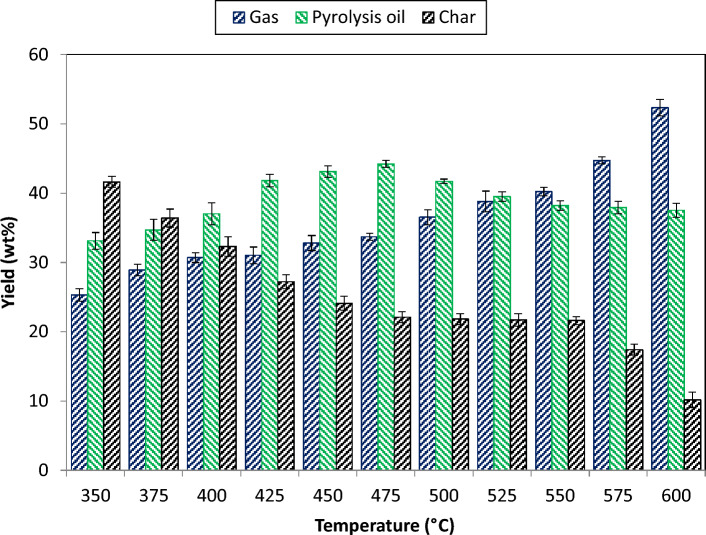
Pyrolysis product distributions.

The impact of reactor temperature on pyrolysis yield distributions is displayed in Fig. 4. The temperature was changed in increasing pattern from 350 to 600 °C to assess the product yield at the interval of 25 °C. All the experiments were conducted by keeping particle size < 1.0 mm. According to the results, the yield of pyrolysis oil enhanced from 33.1 wt% to 41.7 wt% at 350 to 500 °C and then decreases to 37.5 wt% at 600 °C. This typical nature of the product is depends on several reactions, both primary and secondary, occur during pyrolysis, producing condensable and non-condensable gaseous products. The condensable gases were further condensed to produce pyrolysis oil. Through the production of non-condensable fragments, secondary processes aid in increasing the gas yield^49^. Inside the reactor, the primary reaction occurs more frequently at lower temperatures and as the reaction temperature increases, more vapour is formed which improves the formation of more condensable volatiles (oil yield). The production of pyrolysis oil, however, decreased after a particular temperature when secondary reactions became more prevalent at a higher temperature^50^. As the reaction temperature rises, more volatiles are formed, which decreases the yield of char. The development of char decreases steadily at elevated temperatures because of the considerable loss of volatiles or secondary breakdown of char. According to Chutia et al.^51^, the secondary breakdown of the primary volatiles also produces certain non-condensable vapours, which further increase the yield of gas. It was also confirmed by the results that when the changed from 350 to 600 °C, the char yield decreased from 41.6 wt% to 10.2 wt% and the gas yield increases from 25.3 wt% to 52.3 wt%. Table 4 shows the tests results of *Madhuca longifolia* pyrolysis in wt% and Table 5 shows mass balance of the yield.

**Table 4:** Tests results of *Madhuca longifolia* pyrolysis in wt%

Case	Temperature in °C	Test 1			Test 2			Test 3		
		Gas	Pyrolysis oil	Char	Gas	Pyrolysis oil	Char	Gas	Pyrolysis oil	Char
Run 1	350	25.0	33.0	42.0	25.7	33.3	41.5	25.3	33.1	41.4
Run 2	375	28.5	34.4	36.2	28.9	34.8	36.5	29.2	34.7	36.5
Run 3	400	30.6	37.0	32.0	30.9	37.4	32.6	30.4	36.8	32.4
Run 4	425	31.2	41.5	27.6	31.0	42.1	27.0	30.8	41.7	27.1
Run 5	450	32.8	43.4	24.0	32.6	43.2	24.1	33.0	42.8	24.2
Run 6	475	33.9	44.2	22.5	34.0	44.2	21.6	33.3	44.1	22.2
Run 7	500	36.6	41.6	21.5	36.6	41.7	22.2	36.4	41.8	21.8
Run 8	525	38.6	39.1	21.4	39.1	39.7	22.1	38.7	39.7	21.7
Run 9	550	40.0	38.0	21.3	40.4	38.4	22.0	40.2	38.2	21.6
Run 10	575	44.3	37.6	17.0	44.9	38.0	18.0	44.9	38.0	17.3
Run 11	600	51.8	37.4	9.60	52.5	37.9	10.4	52.6	37.3	10.5

**Table 5:** Mass balance.

		Unit	Run 1	Run 2	Run 3	Run 4	Run 5	Run 6	Run 7	Run 8	Run 9	Run 10	Run 11
Input	Biomass	Gram	60.00	60.00	60.00	60.00	60.00	60.00	60.00	60.00	60.00	60.00	60.00
Output^a^	gas^b^	Gram	15.18	17.34	18.42	18.60	19.68	20.22	21.90	23.28	24.12	26.82	31.38
	Pyrolysis oil	Gram	19.86	20.82	22.20	25.08	25.86	26.52	25.02	23.70	22.92	22.74	22.50
	Char	Gram	24.96	21.84	19.38	16.32	14.46	13.26	13.08	13.02	12.96	10.44	06.12
Mass in/mass out		–	1.00	1.00	1.00	1.00	1.00	1.00	1.00	1.00	1.00	1.00	1.00

^a^Average from test 1, test 2 and test 3 in Table 4. ^b^Computed by material balance.

### Characteristics of pyrolysis oil

It is important to pay attention to the quality of the pyrolysis oil produced by the pyrolysis process, which is considerably influenced by the pyrolysis temperature. The pyrolysis oil produced at a lower pyrolysis temperature (< 300 °C) generally has higher moisture content and a lower viscosity. In contrast, pyrolysis oil produced at a higher pyrolysis temperature (> 600 °C) has a higher viscosity and lower moisture content^52^. Furthermore, the temperature during pyrolysis can have an impact on the chemical composition of the oil. Pyrolysis oils typically contain higher concentrations of aldehydes and fatty acids at lower temperatures. On the other hand, aromatic substances like catechol and phenol are likely to increase at higher temperatures. In this study, the pyrolysis oil obtained at maximum oil yield conditions (475 °C) was taken for the physical and chemical characterization study.

#### Physical analysis

It was of interest to examine the physical properties of pyrolysis oil since it is very important to describe the application of the produced oil. Table 6 demonstrates the physical characteristics of the produced pyrolysis oil and other pyrolysis oils derived from various feedstocks. The table also compared the physical nature of conventional diesel. The pyrolysis oil fraction had a distinct appearance compared to conventional diesel fuel. The pyrolysis oil was dark brown and more viscous than diesel. In general, the typical pyrolysis oil has water components of about 25 wt% or more. In contrast, the water content of the derived *Madhuca longifolia* oil was less than 20 wt%. Viscosity is a crucial characteristic of pyrolysis oil, which represents the flow ability of any liquid. The higher viscosity of the fuel disturbs the pumping and atomization during burning. Pyrolysis oil is derived from a variety of biomasses under varying operating conditions and has a different range of viscosities. The viscosity of pyrolysis oil derived from *Madhuca longifolia* residues shows 4.0 cSt, which is higher than the value obtained from *Mimusops elengi*, sugarcane leaves, and napier grass. Despite being produced under the same operating conditions, the bio-oil derived from two different feedstocks has different viscosities. For instance, at 50 °C, the viscosity of the pyrolysis oil made from wheat straw, pine, and ensyn was shown to be 11 cSt, 46 cSt, and 50 cSt, respectively^53^. The variation in viscosity is determined by the structure and composition of the parent feedstocks. The density of the pyrolysis oil is found to be another important physical characteristic. The density of the typical pyrolysis oil is found in the range of 1000–1250 kg/m^3^. This variation is primarily caused by the type of biomass used for the pyrolysis process. The density of the *Madhuca longifolia* pyrolysis oil is found to be 995 kg/m^3^ which is less than all pyrolysis oils produced from the various feedstocks listed in Table 6. The water content in the pyrolysis oil has some adverse effects while burning. The presence of water molecules reduces the calorific value of the oil and is also responsible for corrosiveness and instability. The pyrolysis oil produced in this study has a pH value of 4.7, which is consistent with other studies. The lower pH value indicated the existence of acidic chemicals. The higher flash point of the pyrolysis oil (130 °C) indicates that it can be stored safely at room temperature.

**Table 6:** Physical characteristics of *Madhuca longifolia* and other pyrolysis oils.

Property	*Madhuca longifolia* [This study]	*Albizia amara*^11^	*Mimusops elengi* oil^52^	Sugarcane leaves^54^	Napier grass^55^	Waste paper^56^	Diesel	Unit
Appearance	Dark brown	#	Dark brown	Dark brown	Dark brown	#	Yellowish	–
Density	995	1050	1130	1089	1274	1205	855	kg/m^3^
Viscosity	4.0	4.2	1.42	0.69	2.32	20	2.3	cSt
Flash point	130	160	#	#	#	200	57	°C
Odour	Smoky	#	Smoky	#	#	#	Aromatic	–
pH value	4.7	3.6	4.22	2.12	2.3	1.5	–	–
Calorific value	22.27	18.63	18.14	27.39	19.79	13.10	43.6	MJ/kg
Water content	19	#	#	8.26	48.15	#	–	wt%

^#^Not reported.

#### Chemical analysis

**Figure 5:**
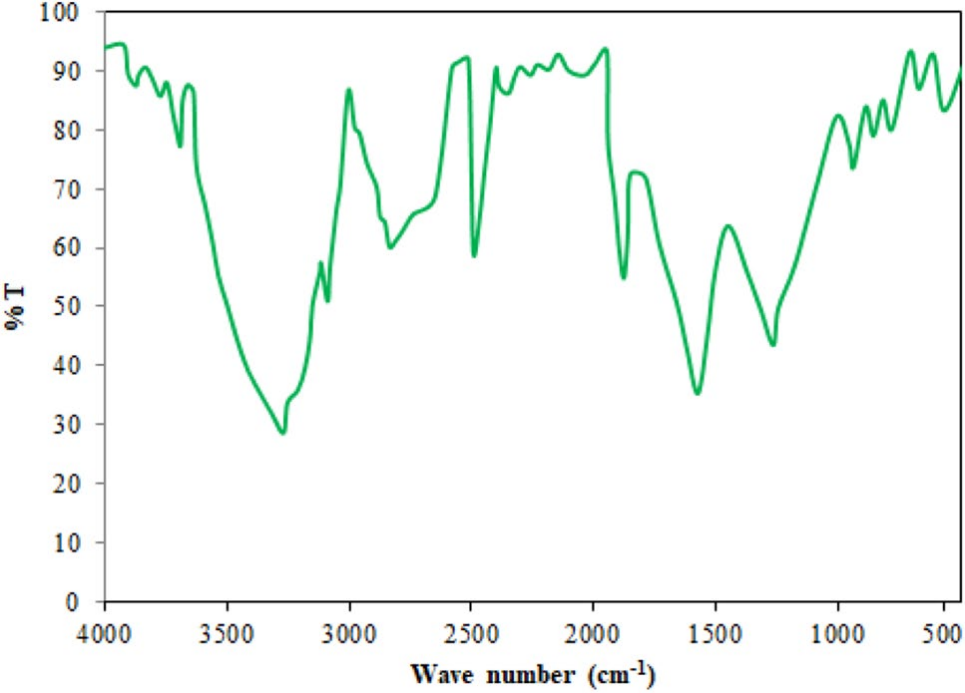
FT-IR analysis of pyrolysis oil.

Figure 5 displays the transmission mode FT-IR spectra for pyrolysis oil between 4000 and 400 cm^−1^. The higher amount of alcohols and phenols is shown by the O–H stretch at 3268.5 cm^−1^. The existence of phenolic or O–H groups holds major percentage in pyrolysis oil^57^. The appearance of alkanes is shown by the C–H stretch at 2835.5 cm^−1^ and 2649.6 cm^−1^. The appearance of alkenes is exposed by the C=C stretch at 1574.7 cm^−1^. The existence of alcohol is shown by the C–O stretch at 942.1 cm^−1^ and the appearance of aromatic compounds is indicated by the C–H bend at 857.8 cm^−1^. The bio-oil revealed an abundance of aliphatic compounds and alcohols. The functional groups of oxygenated C–O and O–H indicate that the pyrolysis oil was significantly hydro-oxygenated, making it naturally acidic. The heating value of pyrolysis oil is reduced when oxygenated functional groups are present. The existence of acids, phenols, alcohols, and aliphatic was also present in different types of pyrolysis oil reported earlier^58,59^. GC–MS analysis can also be used to justify the aforementioned functional groups in pyrolysis oil^57^.

In GC–MS, the volatile matter present in the oil sample is separated and identified using the NIST library. For the analysis, the liquid sample is converted into a vapour which can be carried by a carrier gas (He). The sample is then transported by the gas over a long, thin glass column coated with a chemical. As the vaporized compounds are strapped into the column, they slow down when they interact with the stationary phase. Depending on their individual chemical characteristics, different compounds will take different amounts of time to reach the end of the column. After the separation process, the compounds are moved to the mass spectrometer (MS). The MS acts as a sensor that recognizes the vaporized compounds and mass information. The structural and chemical characteristics of molecules can be identified, measured, and ascertained using mass information. Generally, pyrolysis oil has over 300 different components due to their complexity^60^. This analysis found more than 60 chemicals in the oil, but the peak areas of more than 0.1% are presented in Table 7. The substance found in pyrolysis oil was formed by the cracking lignocellulosic content of the feedstock. Among several chemical groups found in mahua pyrolysis oil, phenolic elements, saturated fatty acids, alkanes, alkenes and branched hydrocarbons were found to be the majority in the oil. The most important substances in the oil are phenols and their derivatives. At the retention times of 11.52 and 30.58 min, phenol and 3,4-dimethylphenol were identified up to an area percentage of 4.94 and 12.25 respectively. Guaiacol occupies the area of 4.58% and Campesterol occupies the area of 3.50%. Guaiacol is a potential component or precursor to green fuels^61^. It is believed that guaiacol provides 85% of the world's vanillin supply. Whereas, campesterol can be used as a precursor for a variety of steroid hormones. 2-hexadecyloxyethanol occupies an area of 4.61% at a retention time of 36.70 min. It is used for various applications, including medicine^62^. Farnesol appears in the pyrolysis oil at a retention time of 32.76 min. Due to its antibacterial properties, farnesol has been used as an organic mite insecticide and as an antiperspirant in cosmetic items. The GC–MS results indicate that derived pyrolysis oil has many fuel-like components, which is suitable replacement for conventional fuel. Apart from that, the compounds identified in the analysis can be separated to be used as feed material for chemical industries.

**Table 7:** GC–MS analysis of the pyrolysis oil.

RT/min	Name of the compounds	Chemical formula	Area %
6.25	2,5-Dimethyl-phenol	C_8_H_10_O	7.21
6.94	o-Cresol	C_7_H_8_O	2.20
8.05	2-Methyl-benzo-furane	C_9_H_8_O	1.25
8.33	Octanoic acid	C_8_H_16_O_2_	2.01
10.08	2-Methoxy-4-methyl-phenol	C_8_H_9_O_2_	2.68
11.52	Phenol	C_6_H_6_O	4.94
13.50	Hexadecane	C_16_H_34_	0.97
14.81	4-Ethyl-2-methoxy-phenol	C_9_H_12_O_2_	3.10
16.07	Propanone	C₃H₆O	2.88
17.00	Butanoic acid	C_4_H_8_O_2_	3.14
17.97	n-Octadecanoic acid	C_18_H_36_O_2_	0.87
19.75	1,103,10 -Terphenyl, 50-phenyl-	C_24_H_18_	3.01
21.22	1,2-Benzenediol	C_6_H_6_O_2_	3.55
21.58	Guaiacol	C_7_H_8_O_2_	4.58
23.41	2,2′-dioxospirilloxanthin	C_42_H_56_O_4_	0.74
26.29	Hydroquinone	C_6_H_6_O_2_	1.99
27.77	3-Methoxy-phenol	C_7_H_8_O_2_	2.24
28.60	γ-Sitosterol	C_29_H_50_O	0.55
29.45	Syringol	C_8_H_10_O_3_	1.58
29.90	9-Tetradecenoic acid	C_14_H_26_O_2_	2.88
30.11	1,3,5 Trimethoxybenzene	C_9_H_12_O_3_	2.97
30.58	Phenol, 3,4-dimethyl-	C_8_H_10_O	12.25
30.60	Chlorodecaborane	C_1_H_13_B_10_	1.18
31.01	Tocopheryl methyl ether	C_29_H_50_O_2_	1.22
31.36	l-Limonene	C_8_H_12_O	2.66
31.92	2-Propanol, 1-(hexadecyloxy)	C_19_H_40_O_2_	1.27
32.28	1,3-Dimethyl-4-azaphenanthrene	C_15_H_13_N	2.55
32.76	Farnesol	C_15_H_26_O	1.81
33.37	Campesterol	C_28_H_48_O	4.50
33.99	3-Hydroxy-2-methylpyridine	C_6_H_7_NO	1.28
34.28	Undecane	C_11_H_24_	2.10
34.50	Octadecanenitrile	C_18_H_35_N	1.08
35.14	Dodecylcyclohexanol	C_18_H_36_O	0.27
35.77	Piperidine-2,5-dione	C_5_H_7_NO_2_	1.29
36.04	2,5-Piperazinedione, 3-benzyl-6-isopropyl-	C_14_H_18_N_2_O_2_	0.63
36.70	2-hexadecyloxy ethanol	C_18_H_38_O_2_	4.61
38.03	2-(2-Isopropenyl-5-methyl-cyclopentyl)-acetamide	C_11_H_19_NO	0.94

During the thermal pyrolysis process, the components of biomass decompose at different rates and with different reaction processes since the reaction is complex and partially dependent on reactor designs and thermal processing parameters. Many studies have previously verified the interactions between the lignocellulosic content of the woody biomass^63^. Hemicellulose and lignin react with each other during pyrolysis to increase the formation of phenolic compounds produced from lignin and inhibit the production of hydrocarbons. Lignin has a major impact on cellulose during pyrolysis since it prevents levoglucosan polymerization, which lowers the formation of char. On the other hand, the reaction between cellulose and hemicellulose has less of an impact on the production and dispersion of pyrolysis products. The primary phase of cellulose pyrolysis is a series of disintegration and polymerization processes that take place at temperatures below 300 °C and form lower molecular components such as furan, hydroxyl acetaldehyde, glycoaldehyde, and formic acid. The main component of pyrolysis vapor is often anhydrosugars, primarily levoglucosan, which are produced by the breaking of glycosidic linkages and dehydrating processes^64^. Above 300 °C, levoglucosan endures relocation and hydration, which produces levoglucosenone. Further cyclization reactions resulted in the formation of stable oxygenated chemicals. The furanic compounds present in the pyrolysis oil are obtained from hemicellulose-based pyrolysis. These compounds were produced due to the dehydration of reducing sugars. The mannose and galactose present in the hemicellulose endure a dehydration reaction to form the hydroxymethyl group^65^. The production of phenol, cresol, guaiacol, and syringol are the primary results of lignin pyrolysis. Guaiacol has two distinct types of carbon–oxygen linkages, which are present in most of the lignin-derived compounds.

### Characteristics of char

#### FT-IR analysis

**Figure 6:**
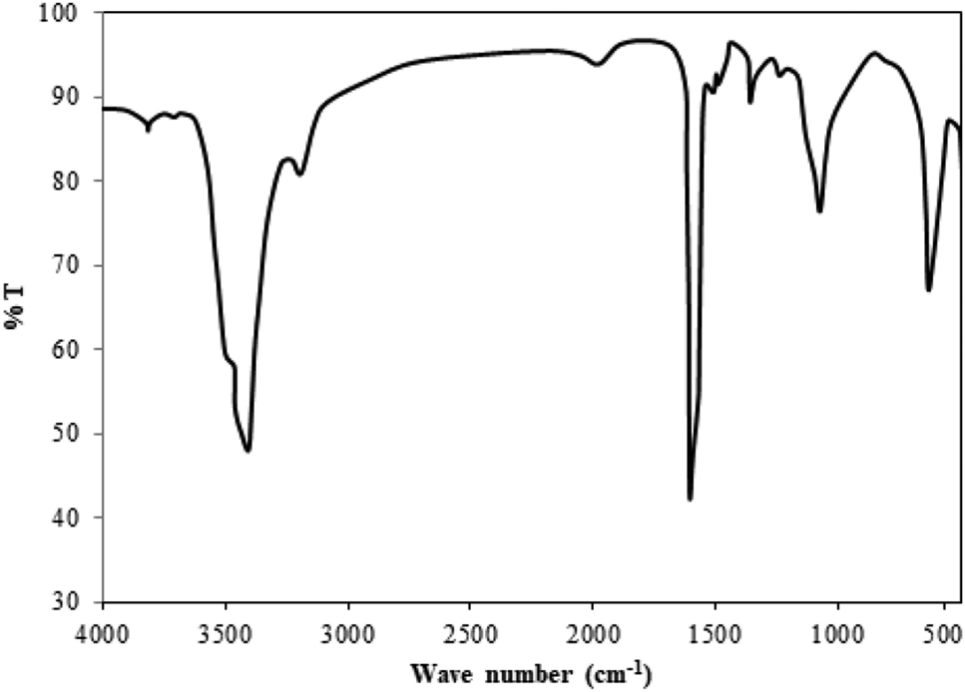
FT-IR spectra of char.

FT-IR spectral characteristics of char are displayed in Fig. 6. The spectra were captured between 4000 and 400 cm^−1^. There could be an acidic or alcoholic composition causing the strong O–H bond stretching vibration at 3404.7 cm^−1^^62^. The vibration at 1605.2 cm^−1^ is indicative of the carboxylic acid in the char^66^. There are alkynes, aliphatic, ketonic, esters and aromatic compounds within the char, which were recognized by the peaks appearing at 1977.6, 1359.4 and 1075.4 cm^−1^ respectively. According to Brodowski et al.^67^, the reactive functional groups present in char, likely O-containing carboxylic, are associated with the broad sensitive zones that engage with polar organic elements and mineral phases. As a result, porous char has oxygen groups that may be good for soil in order to enhance its physical characteristics.

#### Chemical composition

Table 8 displays the approximate composition present in char. Raw feedstock was converted into char, which led to decreased volatile and moisture contents with increased fixed carbon and ash fractions. It has been suggested that the fixed carbon in char is naturally resilient to biological degradation. Generally, pyrolysis char has higher fixed carbon and lower volatile matters. The ash present in the char is cautiously attractive if it is used in soil for agronomy and carbon sequestration. The pyrolysis char has fixed carbon content of 52.7% and volatile matter and ash of 28.85% and 16.14%, respectively. Elemental analysis demonstrates that the pyrolysis of biomass increased carbon content while decreasing hydrogen, oxygen and sulfur in char compared to feedstock material (Table 9). Pyrolysis of *Madhuca longifolia* residues modified the constituent carbon molecules to produce char with higher aromatic carbon and less hydrogen and oxygen^68^. The decrease in oxygen-containing species in char may be due to dehydration and the decarboxylation process. Sulfur content in char may have decreased as a result of organic sulfur decomposition at elevated temperatures. The H/C and O/C molar ratios were identified as 0.623 and 0.373. It helps to determine the durability of the char against degradation. For char, a half-life of over a 1000 years is anticipated when the O/C ratio is less than 0.2^69^. The volatile matter in char is made up of molecules with greater H/C ratios, while the fixed carbon has a lower hydrogen concentration. The HHV of the char components was identified as 21.2 MJ/kg.

**Table 8:** Proximate analysis of char.

Component	Moisture	Ash	Volatile matter	Fixed carbon^a^
Value in %	2.31	16.14	28.85	52.7

^a^By difference.

**Table 9:** Elements of char.

Component	C	H	N	S	O^a^	H/C	O/C	HHV
Value in %	60.4	3.3	0.9	1.3	34.1	0.623	0.373	21.2

^a^By difference.

### Characteristics of gas

Table 10 shows the elements of the non-condensable gas fractions. The finding shows a variety of combustible hydrocarbons. The components were identified as CH_4_, CO_2_, CO, O_2_ and H_2_ in different ratios. An average concentration of 33.1% of CO_2_ was found in the gas, along with 6.8% of CO. Increased CO_2_ production is primarily caused by the water–gas shifting process of CO and breakdown of C–O and –COOH groups^69^. According to Sowmya Dhanalakshmi and Madhu^70^, the reverse Boudouard process amongst char and produced CO_2_ might be the cause of the increased CO level. According to Tinwala et al.^71^, the identification of CO and CH_4_ in pyrolysis gas is the outcome of the secondary cracking of produced volatiles and the production of higher CO_2_ is also caused by the deprivation of cellulosic content. This increased CO_2_ percentage suggests that the *Madhuca longifolia* residues contain oxygenated elements and it is noteworthy that the concentration of CO in the pyrolysis gas rose at higher temperatures due to secondary cracking process. The observations revealed that the pyrolysis gas contains 17.3% flammable methane. Methane is formed at medium and elevated pyrolysis temperatures due to deterioration of benzyl and methoxyl groups. On the other side, the formation of hydrogen is due to the hydrolysis of aromatic components.

**Table 10:** Averaged gas composition of pyrolysis gas.

Component	H_2_	O_2_	CO	CH_4_	CO_2_	N_2_
Value in %	2.6	10.9	6.8	17.3	33.1	25.5

## Applications of pyrolysis product yields

Biomass can be a valuable resource for the pyrolysis process, providing a variety possibilities to increase its added value and produce bioproducts that are in high demand. Pyrolysis oil, or bio-oil, char, and gas are the three main products derived from the pyrolysis process. Nowadays, the usefulness of pyrolysis oils as a renewable fuel is gaining more interest. The raw pyrolysis oil can be used as a low-grade fuel for furnaces and boilers. The pyrolysis oils are generally more difficult to combine with synthetic fuels derived from petroleum. However, the substances derived from pyrolysis oils have a wide range of applications in the medical, cosmetic, and pharmaceutical industries. The oil contains phenols and their derivatives, which are frequently utilized in food preparation, transport, and colorants^72^. The fatty acid composition present in the oil can potentially be utilized for producing natural pesticides. Anyhow, the pyrolysis oil made from *Madhuca longifolia* residues eventually replaces fossil asphalt because of its considerable heating value. The calorific value of the oil indicates that it is almost 40% more than that of diesel fuel, making it suitable for forestry residues. So the produced pyrolysis oil can be used as a low-grade fuel for furnaces and boilers. The char produced in this study can be used as a substitute for traditional solid fuels. Further study is needed to convert the produced char into activated carbon. However, it can be compressed into charcoal briquettes or used to make gunpowder. The utilization of char is largely contingent upon the composition of the material to be pyrolyzed. Due to the higher surface area and porosity of the char, it is most frequently used as an adsorbent material. The pyrolysis gas can also be used as a source for producing heat for various heating processes. The produced pyrolysis gas is a mixture of intriguing molecules that includes considerable amounts of H_2_, CH_4_, and higher hydrocarbons. Moreover, post-treatment might increase the amount of these molecules, making them a valuable source of biomolecules.

## Challenges and opportunities

The pyrolysis oil comprises hydrocarbons, oxygenated compounds, and nitrogenated compounds, as indicated in Table 3. Compounds containing oxygen have the potential to lower its calorific value, stability, and flow ability. The existence of nitrogenated compounds can lead to NOx emissions during combustion. So it is very essential to improve the hydrocarbon ratios in pyrolysis oil. The extraction of oxygenated compounds from pyrolysis can be done by various pyrolysis steps, including dehydration, decarbonylation, decarboxylation, and hydrodeoxygenation; however, studies on the extraction of nitrogenated compounds from pyrolysis oil are rare. According to Li et al.^73^, selective adsorption or hydrodenitrogenation can be used to remove nitrogenated compounds. These methods can also ensure the pyrolysis oil has a better calorific value, higher hydrocarbon content, higher stability, and a lower viscosity. Before solving the pyrolysis task, researchers need to think about the examination of pyrolysis mechanisms. To enhance current global kinetic systems, new experimental methods with molecular-level understanding are required. During pyrolysis, the feedstock is heated, and the produced volatiles are collected and quantified. But the characterization of liquid- and solid-phase intermediates was generally omitted as a result of their shorter lifespans and complexity. Therefore, the absence of experimental data related to condensed-phase intermediates may simplify the development of liquid- and solid-phase reaction mechanisms. It is also obvious that the prediction of heat profiles of feedstock inside the reactor is difficult due to the lack of experimental data. So, further study is needed to analyze the heat transfer phenomena of the lignocellulosic particle. In order to increase the production of pyrolysis oil from *Madhuca longifolia* residues, catalytic pyrolysis have been suggested on commercial and laboratory scales. Instead of a fixed-bed reactor, the residues can be pyrolyzed in other types of reactors, such as fluidized-bed reactors, microwave-assisted reactors, ablative reactors, etc., to improve the selective product output. Based on the research, an optimization study is also recommended.

## Conclusion

Pyrolysis is a potential choice to produce alternative fuel by utilizing forestry residues. *Madhuca longifolia* residues contain a higher percentage of volatile matters (71.26%), lower moisture (5.98%), ash (4.50%), and sulfur (1.20%) were pyrolyzed in a batch-type fixed-bed reactor at temperatures between 350 and 600 °C. The products produced from the pyrolysis were analyzed using several analytical characterization techniques. The variations in product yields have been noted between different operating temperatures, and the maximum pyrolysis oil yield of 44.2 wt% was found at a temperature of 475 °C. The gaseous yield continuously developed, whereas the char yield reduced as the temperature exceeded 475 °C. The FT-IR analysis of the pyrolysis soil showed the presence of different functional groups such as O–H, C=O, C–H and C=C. The GC–MS analysis of the pyrolysis oil showed the existence of different major chemicals such as 3,4-dimethylphenol, 2,5-dimethylphenol, phenol, guaiacol, 2-hexadecyloxy ethanol, campesterol and butanoic acid. The physicochemical characteristics of the pyrolysis oil show that the presence of water-based molecules and its higher viscosity restrict its direct usage in furnaces and engines; however, it can be used as a low-grade fuel and can be upgraded for further usage. The potential applications of pyrolysis char as an adsorbent and biofertilizer were revealed by the qualitative examination. Gas chromatography results of the pyrolysis gas demonstrated its use as gaseous fuel by confirming the presence of combustible components.

## Data Availability

The data generated or analyzed during this study are available within the article.

## References

[1] Mohan I, Panda AK, Volli V, Kumar S (2024). An insight on upgrading of biomass pyrolysis products and utilization: Current status and future prospect of biomass in India. Biomass Convers. Biorefin..

[2] Rahman MM, Aravindakshan S, Matin MA (2021). Design and performance evaluation of an inclined nozzle and combustor of a downdraft moving bed gasifier for tar reduction. Renew. Energy.

[3] Kumar G, Dharmaraja J, Arvindnarayan S, Shoban S, Bakonyi P, Saratale GD, Kim SH (2019). A comprehensive review on thermochemical, biological, biochemical and hybrid conversion methods of bio-derived lignocellulosic molecules into renewable fuels. Fuel.

[4] Rahman MM, Henriksen UB, Ahrenfeldt J, Arnavat MP (2020). Design, construction and operation of a low-tar biomass (LTB) gasifier for power applications. Energy.

[5] Seah CC, Tan CH, Arifin NA, Hafriz RSRM, Salmiaton A, Nomanbhay S, Shamsuddin AH (2023). Co-pyrolysis of biomass and plastic: Circularity of wastes and comprehensive review of synergistic mechanism. Results Eng..

[6] Gupta A, Chaudhary R, Sharma S (2012). Potential applications of mahua (*Madhuca indica*) biomass. Waste Biomass Valoriz..

[7] Mishra RK, Mohanty K (2020). Pyrolysis characteristics, fuel properties, and compositional study of *Madhuca longifolia* seeds over metal oxide catalysts. Biomass Convers. Biorefin..

[8] Raj R, Singh DK, Tirkey JV (2022). Co-gasification of Low-grade coal with *Madhuca longifolia* (Mahua) biomass and dual-fuelled mode engine performance: Effect of biomass blend and engine operating condition. Energy Convers. Manag..

[9] Shanmuga Priya M, Rajalakshmi R (2023). Comparison of porous carbon electrodes derived from *Madhuca longifolia* leaves by hydrothermal technique and direct pyrolysis techniques. Asian J. Chem..

[10] Madhu P, Vidhya L, Vinodha S, Wilson S, Sekar S, Patil PP, Prabhakar S (2022). Co-Pyrolysis of hardwood combined with industrial pressed oil cake and agricultural residues for enhanced bio-oil production. J. Chem..

[11] Rahman MM, Henriksen UB, Ciolkosz D (2023). Startup process, safety and risk assessment of biomass gasification for off-grid rural electrification. Sci. Rep..

[12] Anandaram H, Srivastava BK, Vijayakumar B, Madhu P, Depoures MV, Patil PP, Prabhakar S (2012). Co-pyrolysis characteristics and synergistic interaction of waste polyethylene terephthalate and woody biomass towards bio-oil production. J. Chem..

[13] Biswas B, Pandey N, Bisht Y, Singh R, Kumar J, Bhaskar T (2017). Pyrolysis of agricultural biomass residues: Comparative study of corn cob, wheat straw, rice straw and rice husk. Bioresour. Technol..

[14] Moreira R, dos Reis Orsini, R., Vaz, J. M., Penteado, J. C. & Spinacé, E. V. (2017). Production of biochar, bio-oil and synthesis gas from cashew nut shell by slow pyrolysis. Waste Biomass Valoriz..

[15] Durak H (2016). Pyrolysis of *Xanthium strumarium* in a fixed bed reactor: Effects of boron catalysts and pyrolysis parameters on product yields and character. Energy Sources Part A..

[16] Alagu RM, Sundaram EG, Natarajan E (2015). Thermal and catalytic slow pyrolysis of *Calophyllum inophyllum* fruit shell. Bioresour. Technol..

[17] Aysu T (2015). Catalytic pyrolysis of *Eremurus spectabilis* for bio-oil production in a fixed-bed reactor: Effects of pyrolysis parameters on product yields and character. Fuel Process. Technol..

[18] Chouhan APS (2015). A slow pyrolysis of cotton stalk (*Gossypium arboretum*) waste for bio-oil production. J. Pharm. Chem. Biol. Sci..

[19] Mohammad I, Abakr Y, Kabir F, Yusuf S, Alshareef I, Chin S (2015). Pyrolysis of Napier grass in a fixed bed reactor: Effect of operating conditions on product yields and characteristics. BioResources.

[20] Aysu T, Durak H, Güner S, Bengü AŞ, Esim N (2016). Bio-oil production via catalytic pyrolysis of *Anchusa azurea*: Effects of operating conditions on product yields and chromatographic characterization. Bioresour. Technol..

[21] Aysu T, Küçük MM (2014). Biomass pyrolysis in a fixed-bed reactor: Effects of pyrolysis parameters on product yields and characterization of products. Energy.

[22] Park J, Lee Y, Ryu C, Park YK (2014). Slow pyrolysis of rice straw: Analysis of products properties, carbon and energy yields. Bioresour. Technol..

[23] Lee Y, Ryu C, Park YK, Jung JH, Hyun S (2013). Characteristics of biochar produced from slow pyrolysis of Geodae-Uksae 1. Bioresour. Technol..

[24] Garg R, Anand N, Kumar D (2016). Pyrolysis of babool seeds (*Acacia nilotica*) in a fixed bed reactor and bio-oil characterization. Renew. Energy.

[25] Imam T, Capareda S (2012). Characterization of bio-oil, syn-gas and bio-char from switchgrass pyrolysis at various temperatures. J. Anal. Appl. Pyrolysis.

[26] Volli V, Singh RK (2012). Production of bio-oil from de-oiled cakes by thermal pyrolysis. Fuel.

[27] Lee MK, Tsai WT, Tsai YL, Lin SH (2010). Pyrolysis of napier grass in an induction-heating reactor. J. Anal. Appl. Pyrolysis.

[28] Pütün AE, Uzun BB, Apaydin E, Pütün E (2005). Bio-oil from olive oil industry wastes: Pyrolysis of olive residue under different conditions. Fuel Process. Technol..

[29] Puy N, Murillo R, Navarro MV, López JM, Rieradevall J, Fowler G, Mastral AM (2011). Valorisation of forestry waste by pyrolysis in an auger reactor. Waste Manag..

[30] Shikha FS, Rahman MM, Sultana N, Mottalib MA, Yasmin M (2023). Effects of biochar and biofertilizer on groundnut production: A perspective for environmental sustainability in Bangladesh. Carbon Res..

[31] Carrasco JL, Gunukula S, Boateng AA, Mullen CA, DeSisto WJ, Wheeler MC (2017). Pyrolysis of forest residues: An approach to techno-economics for bio-fuel production. Fuel.

[32] Amutio M, Lopez G, Alvarez J, Moreira R, Duarte G, Nunes J, Bilbao J (2013). Flash pyrolysis of forestry residues from the Portuguese Central Inland Region within the framework of the BioREFINA-Ter project. Bioresour. Technol..

[33] Charon N, Ponthus J, Espinat D, Broust F, Volle G, Valette J, Meier D (2015). Multi-technique characterization of fast pyrolysis oils. J. Anal. Appl. Pyrolysis.

[34] Schellekens J, Silva CA, Buurman P, Rittl TF, Domingues RR, Justi M, Trugilho PF (2018). Molecular characterization of biochar from five Brazilian agricultural residues obtained at different charring temperatures. J. Anal. Appl. Pyrolysis.

[35] Papari S, Hawboldt K (2018). A review on condensing system for biomass pyrolysis process. Fuel Process. Technol..

[36] Hu Y, Stevens DM, Man S, Crist RM, Clogston JD (2019). Total drug quantification in prodrugs using an automated elemental analyzer. Drug Deliv. Transl. Res..

[37] Oyedun AO, Tee CZ, Hanson S, Hui CW (2014). Thermogravimetric analysis of the pyrolysis characteristics and kinetics of plastics and biomass blends. Fuel Process. Technol..

[38] Coates J (2000). Interpretation of infrared spectra, a practical approach. Encycl. Anal. Chem..

[39] McKendry P (2022). Energy production from biomass (part 1): Overview of biomass. Bioresour. Technol..

[40] Sowmya Dhanalakshmi C, Kaliappan S, Mohammed Ali H, Sekar S, Depoures MV, Patil PP, Birhanu HA (2022). Flash pyrolysis experiment on albizia odoratissima biomass under different operating conditions: A comparative study on bio-oil, biochar, and noncondensable gas products. J. Chem..

[41] Rahman MM (2022). Test and performance optimization of nozzle inclination angle and swirl combustor in a low-tar biomass gasifier: A biomass power generation system perspective. Carbon Resour. Convers..

[42] Gray MR, Corcoran WH, Gavalas GR (1985). Pyrolysis of a wood-derived material. Effects of moisture and ash content. Ind. Eng. Chem. Process Des. Dev..

[43] Yildiz G, Ronsse F, Venderbosch R, van Duren R, Kersten SR, Prins W (2015). Effect of biomass ash in catalytic fast pyrolysis of pine wood. Appl. Catal. B..

[44] Dwivedi KK, Karmakar MK, Chatterjee PK (2020). Thermal degradation, characterization and kinetic modeling of different particle size coal through TGA. Therm. Sci. Eng. Prog..

[45] Leng E, Guo Y, Chen J, Liu S, Jiaqiang E, Xue Y (2022). A comprehensive review on lignin pyrolysis: Mechanism, modeling and the effects of inherent metals in biomass. Fuel.

[46] Liew RK, Nam WL, Chong MY, Phang XY, Su MH, Yek PNY, Lam SS (2018). Oil palm waste: An abundant and promising feedstock for microwave pyrolysis conversion into good quality biochar with potential multi-applications. Process. Saf. Environ. Prot..

[47] Mishra RK, Mohanty K (2018). Pyrolysis kinetics and thermal behavior of waste sawdust biomass using thermogravimetric analysis. Bioresour. Technol..

[48] Chintala V, Kumar S, Pandey JK, Sharma AK, Kumar S (2017). Solar thermal pyrolysis of non-edible seeds to biofuels and their feasibility assessment. Energy Convers. Manag..

[49] Kaushik VS, Dhanalakshmi CS, Madhu P, Tamilselvam P (2022). Co-pyrolysis of neem wood bark and low-density polyethylene: Influence of plastic on pyrolysis product distribution and bio-oil characterization. Environ. Sci. Pollut. Res..

[50] Horne PA, Williams PT (1996). Influence of temperature on the products from the flash pyrolysis of biomass. Fuel.

[51] Chutia RS, Kataki R, Bhaskar T (2014). Characterization of liquid and solid product from pyrolysis of *Pongamia glabra* deoiled cake. Bioresour. Technol..

[52] Maulinda L, Husin H, Arahman N, Rosnelly CM, Syukri M, Nurhazanah F, Ahmadi N (2023). The influence of pyrolysis time and temperature on the composition and properties of bio-oil prepared from tanjong leaves (*Mimusops elengi*). Sustainability.

[53] Sipilä K, Kuoppala E, Fagernäs L, Oasmaa A (1998). Characterization of biomass-based flash pyrolysis oils. Biomass Bioenergy.

[54] Kumar M, Upadhyay SN, Mishra PK (2022). Pyrolysis of sugarcane (*Saccharum officinarum* L.) leaves and characterization of products. ACS Omega.

[55] Suntivarakorn R, Treedet W, Singbua P, Teeramaetawat N (2018). Fast pyrolysis from Napier grass for pyrolysis oil production by using circulating Fluidized Bed Reactor: Improvement of pyrolysis system and production cost. Energy Rep..

[56] Islam MN, Beg MRA, Islam MR (2005). Pyrolytic oil from fixed bed pyrolysis of municipal solid waste and its characterization. Renew. Energy.

[57] Bhattacharjee N, Biswas AB (2019). Pyrolysis of orange bagasse: Comparative study and parametric influence on the product yield and their characterization. J. Environ. Chem. Eng..

[58] Biradar CH, Subramanian KA, Dastidar MG (2014). Production and fuel quality upgradation of pyrolytic bio-oil from Jatropha Curcas de-oiled seed cake. Fuel.

[59] Mulimani HV, Navindgi MC (2018). Production and characterization of bio-oil by pyrolysis of Mahua de-oiled seed cake. Chem. Sel..

[60] Lu Q, Li WZ, Zhu XF (2009). Overview of fuel properties of biomass fast pyrolysis oils. Energy Convers. Manag..

[61] Saidi M, Samimi F, Karimipourfard D, Nimmanwudipong T, Gates BC, Rahimpour MR (2014). Upgrading of lignin-derived bio-oils by catalytic hydrodeoxygenation. Energy Environ. Sci..

[62] Wu W, Yang M, Feng Q, McGrouther K, Wang H, Lu H, Chen Y (2012). Chemical characterization of rice straw-derived biochar for soil amendment. Biomass Bioenergy.

[63] Kan T, Strezov V, Evans TJ (2016). Lignocellulosic biomass pyrolysis: A review of product properties and effects of pyrolysis parameters. Renew. Sustain. Energy Rev..

[64] Stefanidis SD, Kalogiannis KG, Iliopoulou EF, Michailof CM, Pilavachi PA, Lappas AA (2014). A study of lignocellulosic biomass pyrolysis via the pyrolysis of cellulose, hemicellulose and lignin. J. Anal. Appl. Pyrolysis.

[65] Yang H, Li S, Liu B, Chen Y, Xiao J, Dong Z, Chen H (2020). Hemicellulose pyrolysis mechanism based on functional group evolutions by two-dimensional perturbation correlation infrared spectroscopy. Fuel.

[66] Yao Y, Gao B, Inyang M, Zimmerman AR, Cao X, Pullammanappallil P, Yang L (2011). Biochar derived from anaerobically digested sugar beet tailings: Characterization and phosphate removal potential. Bioresour. Technol..

[67] Brodowski S, Amelung W, Haumaier L, Abetz C, Zech W (2005). Morphological and chemical properties of black carbon in physical soil fractions as revealed by scanning electron microscopy and energy-dispersive X-ray spectroscopy. Geoderma.

[68] Bian R, Ma B, Zhu X, Wang W, Li L, Joseph S, Pan G (2016). Pyrolysis of crop residues in a mobile bench-scale pyrolyser: product characterization and environmental performance. J. Anal. Appl. Pyrolysis.

[69] Quan C, Xu S, An Y, Liu X (2014). Co-pyrolysis of biomass and coal blend by TG and in a free fall reactor. J. Therm. Anal. Calorim..

[70] Sowmya Dhanalakshmi C, Madhu P (2021). Biofuel production of neem wood bark (*Azadirachta indica*) through flash pyrolysis in a fluidized bed reactor and its chromatographic characterization. Energy Sources Part A..

[71] Tinwala F, Mohanty P, Parmar S, Patel A, Pant KK (2015). Intermediate pyrolysis of agro-industrial biomasses in bench-scale pyrolyser: Product yields and its characterization. Bioresour. Technol..

[72] Negahdar L, Gonzalez-Quiroga A, Otyuskaya D, Toraman HE, Liu L, Jastrzebski JT, Weckhuysen BM (2016). Characterization and comparison of fast pyrolysis bio-oils from pinewood, rapeseed cake, and wheat straw using 13C NMR and comprehensive GC× GC. ACS Sustain. Chem. Eng..

[73] Li F, Srivatsa SC, Bhattacharya S (2019). A review on catalytic pyrolysis of microalgae to high-quality bio-oil with low oxygeneous and nitrogenous compounds. Renew. Sustain. Energy Rev..

